# Factors associated with knowledge, attitudes, and behaviors regarding antiviral medications for COVID-19 among US adults

**DOI:** 10.1371/journal.pone.0335182

**Published:** 2025-11-11

**Authors:** E. Ivy Oyegun, Muyiwa Ategbole, Cynthia Jorgensen, Allison Fisher, Melissa Briggs Hagen, Lindsay Gutekunst, Eric Roberts, Emilia H. Koumans

**Affiliations:** 1 National Center for Immunization and Respiratory Disease, Centers for Disease Control and Prevention, Atlanta, Georgia, United States of America; 2 Tanaq Support Services, Anchorage, Alaska, United States of America; 3 Emory University Rollins School of Public Health, Atlanta, Georgia, United States of America; 4 KRC Research, Washington, DC, United States of America; University of Health and Allied Sciences, GHANA

## Abstract

**Background:**

Little is known about public perceptions of antivirals for the treatment of mild-to-moderate COVID-19 in the United States (US). Our objective was to explore adult perceptions toward COVID-19 antivirals to improve outreach communications about antivirals for COVID-19.

**Methods:**

During July 2022, potential respondents ≥18 years were randomly sampled from a national opt-in internet panel, with oversampling of African Americans, Hispanics, and adults ≥65 years. Respondents were asked about sociodemographic factors, and knowledge, attitudes, and perceptions regarding COVID-19 antivirals. Results were weighted to represent the non-institutionalized US adult population.

**Results:**

Among 1,155 respondents, 51% were female, 21% were 50−64 years, and 19% were ≥65 years. Compared to younger age groups, a greater proportion of adults ≥65 years were knowledgeable about COVID-19 antivirals and would take them if they tested positive or their doctor recommended them. Adults ≥65 years and those reporting immunosuppression or disability had the highest rates of willingness to take antivirals. For all groups, the proportion of people willing to take antivirals increased by >20% if recommended by their doctor. Respondents in the 50−64 and ≥65 groups who were sure they would take COVID-19 antivirals were more likely to be fully vaccinated and less likely to be living in isolation.

**Conclusion:**

Groups that are less likely to have been vaccinated, those living in isolation, and those not sure about whether they would take an antiviral or not may be at risk for not receiving treatment to prevent severe COVID-19 outcomes. However, trust in doctor recommendations may be enough to overcome individual patient concerns about COVID-19 antivirals. Targeted initiatives to educate those at risk for severe COVID-19 outcomes about the effectiveness of antivirals may be needed to further lower this population’s risk of severe COVID-19.

## Background and introduction

In late 2021, the United States (US) Food and Drug Administration (FDA) authorized [1–5] and subsequently the Infectious Diseases Society of America (IDSA) treatment guidelines recommended [6–9] COVID-19 antivirals for adults ≥50 years and for those with underlying medical conditions to reduce the risk for severe outcomes from COVID-19. However, among those eligible only 28%−48% are receiving these medications [10–12]. There are likely a multitude of reasons for this low uptake stemming from both provider and patient perspectives, as well as logistic considerations such as access. Potential contributing factors from the provider perspective for reduced use of antivirals include concerns about the risk of rebound as well as concerns about drug-drug interactions [10,13–16]. While multiple articles have reported on disparities in COVID-19 treatment use, with significant differences reported by race/ethnicity and socio-economic status, there has been inadequate exploration of the reasons for these disparities or the interventions required to address them [17,18].

Public perception about the use of COVID-19 antivirals was poorly understood in 2022 [13,19–21]. Understanding psycho-social determinants of health such as knowledge, attitudes, and perceptions (KAPs) have proven useful in identifying potential underlying misconceptions and barriers hindering patient uptake of treatment, and in shaping targeted strategies for successful adoption and subsequently improved management of diseases [22,23]. If knowledge about COVID-19 antivirals among those eligible is low, their full benefit may not be realized [24]. In the same vein, availability of antivirals is enhanced by belief in their efficacy and effectiveness for successful uptake [25]. As such it was important to assess the underlying gaps in public knowledge, attitudes, and perceptions to better understand and identify opportunities and methods to improve antiviral uptake. While literature exists on the public’s KAPs of COVID-19 stay-at-home orders, vaccinations, and boosters [26,27], there was limited literature on the public’s KAPs towards COVID-19 antivirals. The objective of this study was to highlight KAPs ascertained through a rapid online survey in the summer of 2022 as they relate to COVID-19 antivirals, focusing on adult awareness, attitudes, and perceived willingness to take the antivirals, with the overall goal of identifying characteristics of populations for enhanced and targeted communications.

## Methods

### Sample

Between July 27, 2022 and July 31, 2022 KRC Research conducted an online cross-sectional survey of U.S. adults aged 18 or older. This 5-minute survey aimed to understand attitudes and behaviors regarding antiviral medications for COVID-19. This survey was funded through a contract between the National Center for Immunizations and Respiratory Diseases Office of Communications and KRC Research. It underwent review by CDC, was deemed research not involving human subjects, and was conducted consistent with applicable federal law and CDC policy (See, e.g., 45 C.F.R. part 46; 21 C.F.R. part 56; 42 U.S.C. §241(d), 5 U.S.C. §552a, 44 U.S.C. §3501 et seq.). Since it was determined that this survey did not involve human subjects under 45 CFR 46, formal consent was inapplicable. Respondents who proceeded with the online survey were considered to have consented to the survey. The survey utilized non-probability sampling drawn from large national panels of participants who have access to the Internet and who opt-in to participate in periodic online sample surveys. Each survey was conducted in English. To ensure demographic representation for analyses, respondents who identified themselves as African American or Black, Hispanic, or were over the age of 65 were oversampled until there were at least 200 respondents within each of those demographic segments. The completion rate of those who chose to participate was 85%. Results were weighted to represent the non-institutionalized US adult population using the 2020 census [26]. All data were fully anonymized before authors received them. Though self-reporting can be accompanied by social desirability bias overestimation of positive behaviors, self-report surveys can have reduced social desirability bias especially if they are anonymous [28–30] as was the case in this study.

### Survey

The following demographic variables were collected from each respondent, response options are included in parenthesis: age (free-text entry 18 years and older included), race (White, Black or African-American, Asian, Other, multi-response answer), ethnicity [Hispanic/Spanish/Latino descent] (yes, no), fully vaccinated status (yes, no), first and second booster status (yes, no), sex (male, female), education level (grade school or less, some high school, high school graduate, some college, 2-year college/technical school, 4-year college, some postgraduate work, postgraduate degree), state of residence (pre-filled drop down menu of all 50 states and D.C.), zip code (5-digit free text), residential community (urban, suburban, rural), home ownership status (own, rent, live with others at no cost), number of adults and children in household (pre-filled drop down menu 0–10), employment status (work full-time, work part-time, self-employed, student, homemaker, retired, omitted, unemployed and seeking work, unemployed and not seeking work, unable to work because of a disability), income level (less than $25,000, $25,000-$29,999, $30,000-$34,999, $35,000-$39,999, $40,000-$49,999, $50,000-$59,999, $60,000-$74,999, $75,000-$99,999, $100,000-$124,999, $125,000-$149,999, $150,000-$199,999, $200,000 or more), political affiliation (Republican, Democrat, independent), immunocompromised status (yes, no, not sure). States were grouped according to the four census regions: Northeast, Midwest, South, West. In-depth questions about underlying medical conditions were not asked.

### Definitions

We stratified the study population into four mutually exclusive groups that have different risks for severe COVID-19 based on age and immunosuppression or disability for the outcome analyses [8,9]. These groups were: adults 18–49 years with no immunosuppression or disability (18–49), adults 50–64 years with no immunosuppression or disability (50–64), adults ≥65 years with no immunosuppression or disability (≥ 65), and adults aged ≥ 18 and older who have immunosuppression or disability (Immunosuppressed or Disabled) [9].

### Outcomes

Respondents were asked to select their level of agreement with questions measuring their knowledge, attitudes, perceptions, preferences, and intentions about COVID-19 antivirals. For each question, the respondent was able to select a variety of graduated responses. Survey questions analyzed for this study contributed to one of three primary outcomes: 1) knowledge of antivirals, 2) attitudes towards antivirals, and 3) perceived willingness to take antivirals. Each of the two questions for outcomes 2 and 3 were analyzed separately. Each of these analyses was performed separately for each of the four groups defined above [9].

The first outcome, *Knowledge of antivirals,* was measured via the following question “Have you heard of antiviral medications to treat people who get infected with COVID-19?” The response options for this question were “Yes”, “No”, “Not sure”. Discriminant analysis, which predicts outcome probabilities by classifying the predictor variables similarly to logistic regression [31], was conducted to identify the characteristics most strongly associated with knowledge of antivirals and compare those who were knowledgeable to those who were unsure or not knowledgeable. Responses of uncertainty such as “Not Sure” have been interpreted as indecision or a lack of knowledge [31,32]. Since this study’s objective was to identify opportunities for targeted messaging, the “Not Sure” respondents would represent a group that could benefit from targeted messaging. Therefore, the “No” and “Not Sure” responses were combined, and the “Yes” response was used as the reference group.

The second outcome, *Attitudes towards antivirals,* was measured via responses to the following two questions “New antiviral medications are effective in decreasing the seriousness of symptoms from a COVID-19 infection” and “New antiviral medications are effective in reducing a person’s chance of getting hospitalized or dying from COVID-19”. Response options for each question were Strongly agree, Agree, Neither agree nor disagree, Disagree, Strongly disagree. Since parametric analyses are robust enough to use with Likert responses [33,34], multiple linear regression was used to identify the characteristics that were most strongly associated with attitudes towards antivirals and to identify the largest differences between those who expressed belief in efficacy of antivirals vs those who did not, with “Strongly agree” or “Agree” as the reference group.

The last outcome, *Perceived willingness to take antivirals,* was measured via responses to the following two questions “I would take antiviral medications if I tested positive for COVID-19.” and “I would take antiviral medications for a COVID-19 infection if my doctor recommended it” (response options for both questions = Yes, No, Not Sure). Discriminant analysis was used to identify the characteristics that were most strongly associated with perceived willingness to take antivirals and to identify the largest differences between those who were willing vs those who were not willing. Responses were modelled as “Yes” vs “Not Sure”, with “Yes” as the reference group. The “No” response was not included in the analysis because these respondents stated their unwillingness to take antivirals implying a higher level of resistance compared to those who were “Not Sure” [35]. Since the goal of the analysis was to identify groups that might be willing to adjust their behaviors given some intervention, this analysis compared those who were willing vs those who were “Not Sure”.

### Statistical analyses

The initial models for each outcome question included 15 variables (gender, age, vaccination status, region, race/ethnicity, community type, marital status, children in home, education, employment status, home ownership, political party, household income, immunocompromised, serious health problem). Since the goal of the analysis was to generate actionable information for communications activities such as which subgroup to target due to higher or lower knowledge, attitude, or willingness, precision about differences between subgroups in each demographic category were not prioritized. Therefore, multiple linear regression or discriminant analysis, as appropriate, were used to identify which social and demographic characteristics were most strongly associated with each outcome. We started with the 15 variables and removed, stepwise, those which were not significant at a p ≤ 0.05, largest p first. There were no *a priori* adjustments made, which might have obscured some findings relevant to communications. Results between linear regression/discriminant analyses and logistic regression were similar as can sometimes occur, and choosing an approach can come down to stylistic preferences [36]. For these analyses, linear regression and discriminant analysis were chosen because the format of the output was more aligned with the study’s objective.

Results include beta coefficients (β) which represent how strongly each variable was associated with its corresponding outcome. Larger coefficients represent stronger associations and vice versa. The “+” sign indicates that respondents with that characteristic where more likely to agree with the corresponding outcome statement, while the “-“sign indicates that respondents with that characteristic where less likely to agree with the corresponding outcome statement.

The strength of each characteristic’s association may vary for each outcome. Therefore, to ensure each outcome had multiple options for communication strategies while focusing on the most associated characteristics, presentation of results highlight the characteristics most strongly associated with each outcome (knowledge, attitudes, and willingness) above a β = +/- 0.2.

## Results

### Demographics

The study population consisted of 1,155 respondents aged ≥18 years. Table 1 displays characteristics of respondents by age and risk groups. Of the respondents, 588 (51%) were female, 692 (60%) were 18–49 years, 240 (21%) were 50–64 years, and 223 (19%) were aged ≥65 years.

**Table 1:** Characteristics of adult survey respondents about knowledge, attitudes, and willingness to take COVID-19 antivirals, weighted %, by age group and immunosuppressed or disability status, US, July 2022^a,b^.

		Non-Immunosuppressed and non-Disabled			Immunosuppressed or Disabled
		18-49 (n = 421) %	50-64 (n = 124) %	≥ 65 (n = 115) %	Immunosuppressed or Disabled (n = 366) %
Total		36.0	11.0	10.0	32.0
Sex	Women	52.0	45.0	47.0	43.0
	Men	47.0	55.0	53.0	57.0
Age (years)	18-24	22.0	0.0	0.0	6.0
	25-34	33.0	0.0	0.0	15.0
	35-44	33.0	0.0	0.0	13.0
	45-54	13.0	35.0	0.0	17.0
	55-64	0.0	65.0	0.0	22.0
	65 or older	0.0	0.0	100.0	27.0
Region^c^	Northeast	16.0	21.0	18.0	18.0
	Midwest	22.0	18.0	19.0	22.0
	South	37.0	36.0	42.0	37.0
	West	25.0	25.0	21.0	23.0
Race/ Ethnicity	Non-Hispanic White	65.0	75.0	85.0	79.0
	Non-Hispanic Black	20.0	10.0	9.0	12.0
	Hispanic	22.0	15.0	4.0	16.0
Community type	Urban	36.0	17.0	20.0	35.0
	Suburban	45.0	61.0	57.0	41.0
	Rural	19.0	21.0	23.0	24.0
Marital Status	Married	34.0	50.0	65.0	41.0
	Not married	66.0	50.0	35.0	59.0
Children in home	Children	49.0	22.0	3.0	29.0
	No children	51.0	78.0	97.0	71.0
Education^d^	High school or less	39.0	35.0	27.0	42.0
	Some college/trade	24.0	26.0	26.0	27.0
	College grad or more	38.0	40.0	47.0	31.0
Employment status^e^	Employed	74.0	68.0	16.0	43.0
	Retired	1.0	15.0	81.0	29.0
	Student	5.0	0.0	0.0	1.0
	Homemaker	7.0	10.0	1.0	4.0
	Not employed currently/unable to work	11.0	1.0	0.0	27.0
Home ownership	Own	41.0	69.0	89.0	49.0
	Rent	48.0	31.0	11.0	45.0
	Live with others at no cost	11.0	1.0	0.0	5.0
Political Party	Democrats & Ind. Leaners	51.0	48.0	49.0	48.0
	Republicans & Ind. Leaners	31.0	36.0	40.0	32.0
	Independent/others do not lean	17.0	15.0	11.0	19.0
Household Income^f^	Less than $35k	34.0	30.0	25.0	46.0
	$35k to less than $50k	13.0	14.0	14.0	10.0
	$50k to less than 75k	18.0	16.0	18.0	17.0
	$75k to less than $100k	12.0	11.0	14.0	5.0
	$100k or more	23.0	29.0	29.0	22.0
Immunocompromised	Yes	0.0	0.0	0.0	100.0
	No	46.0	18.0	15.0	21.0
Serious Health Problem	Yes	0.0	0.0	0.0	100.0
	No	53.0	21.0	18.0	8.0
Vaccination status	Fully vaccinated	21.0	26.0	28.0	25.0
	Received First Booster	29.0	15.0	17.0	40.0
	Received Second Booster	0.0	15.0	26.0	59.0

^a^Column percentages are displayed here; Only includes respondents with complete demographic data.

^b^Results were weighted to represent the non- institutionalized US adult population using the 2020 census.

^c^The 50 states and D.C. were grouped into these 4 Census regions.

^d^Education levels were collapsed as follows: High School or less= Grade school or less, Some high school, High school graduate; Some college/trade = Some college, 2-year college/technical school; College graduate or more= 4-year college, Some postgraduate work, Postgraduate degree.

^e^Employed includes: Work full-time , Work part-time, and Self-employed; Not employed currently/unable to work includes: Unemployed and seeking work, Unemployed and not seeking work, Unable to work because of a disability.

^f^Income levels were collapsed as follows: Less than $ 35k includes Less than $ 25,000, $ 25,000- $ 29,999, and $ 30,000- $ 34,999; $ 35k to less than $ 50k includes $ 35,000- $ 39,999 and $ 40,000- $ 49,999; $ 50k to less than $ 75k includes $ 50,000- $ 59,999 and $ 60,000- $ 74,999; $ 75k to less than $ 100k includes $ 75,000- $ 99,999; $ 100k or more includes $ 100,000- $ 124,999, $ 125,000- $ 149,999, $ 150,000- $ 199,999, and $ 200,000 or more.

### Perceptions and willingness to take antivirals

**Fig 1:**
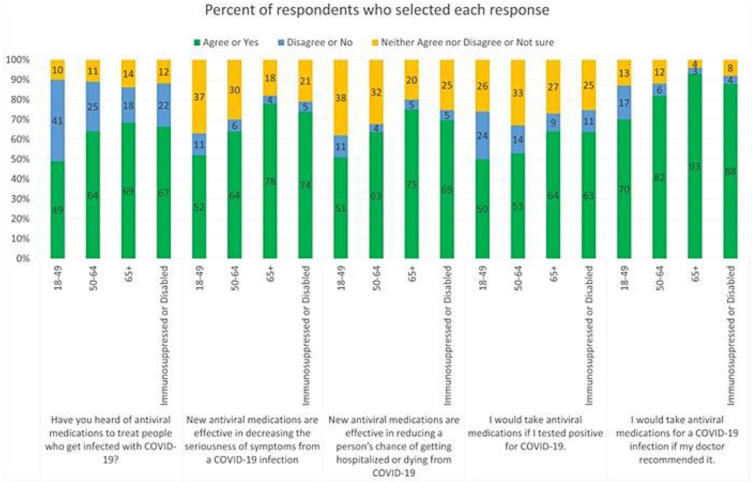
Distribution of respondents’ answers to question about antiviral knowledge, attitudes toward antivirals, and willingness to take antivirals.

The following results are shown in Fig 1 which summarizes knowledge and attitudes and perceptions about antiviral effectiveness and willingness to take them by age and risk group. Respondents ≥65 years represented the highest proportion (69%) who had heard of antiviral medications, followed by the Immunosuppressed or Disabled group (67%). A greater proportion of respondents ≥65 years agreed that antivirals reduced the chances of developing severe outcomes such as serious symptoms (78%) or of being hospitalized/dying from COVID-19 (75%) compared to those aged 50–64 (64%, 63% respectively) and those aged 18–49 (52%, 52% respectively). This was slightly higher than among respondents that were immunosuppressed or disabled (74%, 69% respectively). When asked about willingness to take antivirals, a greater proportion of respondents ≥65 years would take antivirals if they tested positive for COVID-19 (64%), or if their doctor recommended it (93%) compared to those aged 50–64 (53%, 82% respectively) and those aged 18–49 (50%, 70% respectively); this was again slightly higher than responses among those who were immunosuppressed or disabled (63%, 88% respectively).

The results below highlight the three characteristics most strongly associated with each outcome (knowledge, attitudes, and willingness) with β > |0.2|. Additional data on model characteristics is presented in Tables 2–4.

**Table 2:** Characteristics associated with knowledge of antivirals among participants 18-49 years old, 50-64 years old, 65 and older, and those with disability or immunosuppression (any age): YES vs NO + NOT SURE.

	18-49 (N = 421)	50-64 (N = 124)	≥ 65 (N = 115)	Immunosuppressed or Disabled (N = 366)
Estimated percent of variance accounted for by the model	9.3%	15.1%	38.7%	14.9%
**Characteristic** ^a^	Statistic (β) ^**b**^	Statistic (β)^**b**^	Statistic (β) ^**b**^	Statistic (β) ^**b**^
Higher level of education	0.484	–	0.632	0.419
Hispanic	–	–	0.432	−0.413
Higher household income	0.551	–	0.591	–
Separated, divorced, widowed	–	−0.508	–	−0.273
Older age^c^	0.44	–	–	–
Boosted	–	–	–	0.374
Live with other adults	–	–	–	0.274
Male	–	–	−0.7	–
Northeast region	–	0.66	–	–
Not working	–	–	–	−0.423
Rural	–	−0.524	–	–
South	–	–	−0.581	–

^a^Discriminant analysis was conducted to identify the characteristics from Table 1 that best predicted knowledge of antivirals and to identify the largest differences between those who were knowledgeable vs those who were not. The “No” and “Not Sure” responses were combined, and the “Yes” response was used as the reference outcome. The referent group for income was ≥ $57,000, for education ≥ 2-year college, and for age, within the 18-49 group, those who were knowledgeable were more likely to be aged 35 to 49 years The referent groups for all other independent characteristics was not having that characteristic, e.g., referent group of “boosted” is “not boosted”. Displayed characteristics account for strongest predictive model. All β values are significant at p=0.05 or less.

^b^The beta coefficients ( ^β^) reported represent how strongly each variable predicted this outcome, while +/- indicates the likelihood that respondents who answered “Yes” to this outcome were more or less likely (respectively) to have the characteristic variable compared to those who answered “No” or “Not Sure”.

^c^Age was subdivided into 18-20, 21-24, 25-29, 30-34. 35-39, 40-44, and 45-49 year old age groups for the 18-49 year age group, 50-54, 55-59, and 60-64 for the 50-64 year old age groups, and 65-69, 70-74, and ≥ 75 for the ≥ 65 year age group.

“- “= adjusted for this characteristic, and not Significant.

**Table 3:** Characteristics associated with attitudes toward antivirals among 18-49 year olds, 50-64 year olds, 65 and older, and those with disability or immunosuppression (any age): 5 point Likert scale from Strongly Agree to Strongly Disagree.

New antiviral medications are effective in decreasing the seriousness of symptoms from a COVID-19 infection: STRONGLY AGREE/AGREE as reference				
	18-49 (n = 374)	50-64 (n = 144)	≥ 65 (n = 127)	Immunosuppressed or Disabled (n = 381)
Estimated percent of variance accounted for	8.3%	13.3%	25.5%	16.2%
Characteristic^a^	Statistic (β) ^b^	Statistic (β) ^b^	Statistic (β) ^b^	Statistic (β) ^b^
Boosted	–	–	0.355	0.155
Older age	0.168	–	–	–
Children 7–12	–	0.166	–	–
Double boosted	–	0.19	–	–
Higher level of education	–	–	–	0.235
Fully vaccinated	0.166	–	–	–
Higher income	–	−0.266	–	–
Male	–	–	−0.259	–
Northeast region	–	0.184	–	–
Not working	–	–	–	−0.113
Republican party or lean	−0.117	–	–	–
Rent a home	−0.125	–	–	–
Retired	–	–	–	0.122
Single	–	0.207	–	–
Urban	–	–	−0.19	–
New antiviral medications are effective in reducing a person’s chance of getting hospitalized or dying from COVID-19: STRONGLY AGREE/AGREE as reference				
	18-49 (n = 373)	50-64 (n = 145)	≥ 65 (n = 127)	Immunosuppressed or Disabled (n = 381)
Estimated percent of variance accounted for	8.8%	21%	36.3%	13.3%
Characteristic^a^	Statistic (β) ^b^	Statistic (β) ^b^	Statistic (β) ^b^	Statistic (β) ^b^
Boosted	0.224	0.204	0.591	0.256
Older age	0.166	0.39	–	0.126
Higher level of education	–	–	0.222	0.144
Male	–	–	−0.198	−0.132
Republican party or lean	−0.12	−0.273	–	–
Children 7–12 in household	–	0.201	–	–

^a^Multiple linear regression was used to identify the characteristics that was most strongly associated with antivirals and to identify the largest differences between those who expressed belief in efficacy of antivirals vs those who did not, with “Strongly agree” or “Agree” as the outcome reference group. The referent groups for income = $ 57,000 +, for education = 2year college +, and for age, those who believed in antiviral effectiveness within the 18-49 group, were more likely to be 35 to 49 years, and within the 50-64 group, were more likely to be 55 to 64 years. The referent groups for all other independent characteristics was not having that characteristic, e.g., referent group of “boosted” is “not boosted”. Displayed characteristics account for strongest predictive model. All ^β^ values are significant at p=0.05 or less.

“- “= not significant.

^b^The beta coefficients ( ^β^) reported represent the strength of the association for this outcome, while +/- indicates the likelihood that respondents who answered “Strongly Agree” to this outcome were more or less likely (respectively) to have the characteristic variable compared to those who did not answer “Strongly Agree”.

**Table 4:** Characteristics associated with willingness to take antivirals among 18-49 year olds, 50-64 year olds, 65 years and older, and those with disability or immunosuppression (any age): Yes vs Not Sure.

Perceived willingness to take anti-viral if test positive: YES (reference) vs NOT SURE				
	18-49 (n = 313)	50-64 (n = 103)	≥ 65 (n = 101)	Immunosuppressed or Disabled (n = 315)
Estimated percent of variance accounted for	6.8%	15.9%	8.5%	12.7%
Characteristic^a^	Statistic (β) ^b^	Statistic (β) ^b^	Statistic (β) ^b^	Statistic (β) ^b^
Boosted	0.606	–	–	0.783
Separated, divorced, widowed	–	−0.596	–	−0.504
Double boosted	–	0.647	–	–
Higher level of education	–	–	0.7	–
Hispanic	0.507	–	–	–
Higher income	–	−0.703	–	–
Living at no cost	–	–	–	−0.373
Own a home	0.722	–	–	–
Western region	–	−0.47	–	–
Work full time	–	–	0.699	–
Perceived willingness to take anti-viral if doctor recommended: YES (reference) vs NOT SURE				
	18-49 no risk (n = 341)	50-64 no risk (n = 115)	≥ 65 no risk (n = 109)	Immunosuppressed or Disabled (n = 338)
Estimated percent of variance accounted for	11%	16.1%	37.3%	15.9%
Characteristic^a^	Statistic (β) ^b^	Statistic (β) ^b^	Statistic (β) ^b^	Statistic (β) ^b^
Fully vaccinated	0.442	0.719	–	0.474
Boosted	–	–	−0.471	0.413
Not working	–	–	0.323	−0.467
Southern region	0.563	−0.605	–	–
Children under 7 in household	–	–	0.718	–
Hispanic ethnicity	0.496	–	–	–
Live with fewer adults	–	–	0.347	–
Own a home	0.542	–	–	–
Single	–	–	0.508	–
Work Part Time or self-employed	0.388	–	–	–

^a^Discriminant analysis was used to identify the characteristics that was most strongly associated with perceived willingness to take antivirals and to identify the largest differences between those who were willing vs those who were not willing. Responses were modelled as “Yes” vs “Not Sure”, with “Yes” as the reference group. Displayed characteristics account for strongest predictive model. All ^β^ values are significant at p=0.05 or less.

^b^The beta coefficients (β) reported represent the strength of the association with this outcome, while +/- indicates the likelihood that respondents who answered “Yes” to this outcome were more or less likely (respectively) to have the characteristic variable compared to those who answered “Not Sure”.

Footnote: “- “= Not Significant.

### Results by group

#### Immunosuppressed or Disabled Group.

In the Immunosuppressed or Disabled group, knowledgeable respondents were more likely to have more education (β = 0.419), and less likely to be non-working (β = −0.423), or Hispanic (β = −0.413) (Table 2).

Respondents who strongly agreed that COVID-19 antivirals could reduce symptom severity were more likely to be better educated (β = 0.235), while respondents who strongly agreed that COVID-19 antivirals could reduce risk of hospitalization or death were more likely to be boosted (β = 0.256) (Table 3).

Respondents who were willing to use antivirals if they tested positive for COVID-19 were more likely to be boosted (β = 0.783) and less likely to be separated, divorced, or widowed (β = −0.504) or to live with others at no cost (β = − 0.373). Conversely, respondents who were willing to use antivirals if their doctor recommended it were more likely to be fully vaccinated (β = 0.474), or boosted (β = 0.413), and less likely to be unemployed (β = −0.467) (Table 4).

#### 65 years and older group.

Knowledgeable respondents in this group were more likely to be educated (β = 0.632) or have higher incomes (β = 0.591). They were also less likely to be male (β = −0.7) (Table 2).

Respondents who strongly agreed that COVID-19 antivirals could reduce symptom severity were more likely to be boosted (β = 0.355), and less likely to be male (β = −0.259). While respondents who strongly agreed that COVID-19 antivirals could reduce risk of hospitalization or death were more likely to be boosted (β = 0.591), or older (β = 0.222) (Table 3).

Respondents who were willing to use antivirals if they tested positive for COVID-19 were more likely to be more educated (β = 0.7) or work full time (β = 0.699). While respondents who were willing to use antivirals if their doctor recommended it were more likely to be fully vaccinated (β = 0.817), or have higher incomes (β = 0.373), and less likely to live with fewer adults (β = −0.587)) (Table 4).

#### 50-64 years group.

Knowledgeable respondents in the 50−64 years age group were more likely to live in the Northeast region (β = 0.66), and less likely to live in a rural setting (β = −0.524), or to be separated, divorced, or widowed (β = 0.508) (Table 2).

Respondents who strongly agreed that COVID-19 antivirals could reduce symptom severity were more likely to have lower incomes (β = −0.266) or be single (β = 0.207). While respondents who strongly agreed that COVID-19 antivirals could reduce risk of hospitalization or death were more likely to be older (β = 0.39), have Democratic leanings (β = −0.273), or boosted (β = 0.204) (Table 3).

Respondents who were willing to use antivirals if they tested positive for COVID-19 were more likely to have lower income (β = −0.703), or be double boosted (β = 0.647), and less likely to be separated, divorced, or widowed (β = −0.596). While respondents who were willing to use antivirals if their doctor recommended it were more likely to be fully vaccinated (β = 0.719), and less likely to live in the Southern region (β = −0.605) (Table 4).

### Thematic results

Compared to lower level of education, higher level of education was a strong predictor of knowledge in the 65 and older, and Immunosuppressed or Disabled groups (β= + 0.632, + 0.419 respectively) with knowledgeable respondents more (+β) likely to be higher educated. Marital status was predictive of knowledge among the 50−64 age group and the Immunosuppressed or Disabled group (β = −0.508, −0.273 respectively), with knowledgeable respondents less (-β) likely to be separated, divorced, or widowed compared to those who were married.

In all study groups, compared to not being boosted, being boosted was a predictor of the belief that antivirals lessen the chance of severe symptoms, hospitalization, or death: 50−64 (β = +0.204), ≥ 65 (β = +0.591), and Immunosuppressed or Disabled (β = +0.256). Marital status was predictive of willingness to take antivirals if a COVID-19 test is positive for the 50−64 (β = −0.596) and the Immunosuppressed or Disabled groups (β = −0.504), with willing respondents less likely (-β) to be separated, divorced, or widowed compared to those who were married.

Compared to not being fully vaccinated, being fully vaccinated was strongly predictive of willingness to take antivirals if their doctor recommended it for all groups except those aged 65 and older: 50–64 (β= + 0.719), Immunosuppressed or Disabled group (β= + 0.474), with willing respondents more likely to be fully vaccinated (+β).

## Discussion

To our knowledge, this is the earliest report of public knowledge, attitudes, and willingness to use antivirals to reduce risk for severe COVID-19 in the United States. This cross-sectional survey, performed approximately 6 months after antivirals became available in the U.S., had diverse representation from each HHS region in the U.S., and was weighted to the non-institutionalized U.S. Census adult population. The proportion of participants willing to take antivirals if recommended was highest (93%) among those aged ≥65 years and those who were immunosuppressed or disabled (88%) and was associated with having received a booster among the latter. While characteristics that were associated with knowledge, attitudes, and willingness to take antivirals differed by age and risk groups, some of these characteristics overlapped. For example, among most groups, higher education was associated with knowledge of antivirals, and among all groups, having had vaccinations or a booster was associated with attitudes toward and willingness to use antivirals. Although the variability explained by the characteristics examined in the models varied, none explained more than 50%, these findings nevertheless have important implications for outreach efforts and communication about COVID-19 antivirals by providing evidence on how to target efforts, for example towards those with lower education and who are unvaccinated, to improve antiviral knowledge and uptake.

### Knowledge

People who are most at risk for severe COVID-19 outcomes, those ≥65 years and those who had a disability or were immunosuppressed, were more likely to know about antivirals if they reported higher levels of income and education. These findings about antiviral knowledge are similar to earlier studies examining knowledge of COVID-19 more generally; these studies also found that socio-demographics, including income and education, were associated with more accurate knowledge of COVID-19 and those with lower education or income levels had less knowledge and were less accurate [37,38]. This suggests that characteristics affecting knowledge which were noted earlier in the pandemic also affect knowledge about antivirals, and that educational efforts about antivirals and risk have been more successful among those with a higher income and education [36]. These findings support the need for additional efforts to inform those with lower income and level of education about COVID-19 risk and about antivirals that address health literacy concerns raised early in the pandemic, and will continue to be relevant as new sub-variants arise [39].

Additional characteristics, such as being separated, divorced, or widowed was associated with being less likely to have COVID-19 antiviral knowledge, while living with other adults and being boosted, were associated with increased knowledge among those at higher risk. These findings suggest that living alone or social isolation is one factor that may negatively affect knowledge [19].

### Attitudes towards antivirals

In all groups examined, we found that having been vaccinated was associated with the beliefs that antivirals reduce the severity of symptoms and that antivirals reduce the likelihood of hospitalization or death. These findings, in combination with the associations of income and education with antiviral knowledge, are similar to studies examining factors associated with vaccination; where people with higher incomes and higher levels of education were more likely to believe in the efficacy of pharmaceuticals and more likely to report acceptance if offered [21].

Other characteristics associated with having a positive attitude toward antivirals preventing hospitalization were older age (within each of the 50−64, ≥ 65, and Immunosuppression or Disability groups), democratic-leaning political affiliation (in the 50−64 group), and being female (in the ≥ 65 and Immunosuppression or Disabled groups). Since the beginning of the pandemic, educational efforts have emphasized that people of older age are at higher risk for severe illness; this finding represents an achievement in risk communication. The finding that political affiliation is associated with attitudes toward antivirals is similar to the finding about attitudes around vaccination [40], where democratic political affiliation has been associated with willingness to get vaccinated. The female sex also played a role in attitudes toward antivirals for those ≥65 years and for those who were immunosuppressed or disabled. The findings around political affiliation and sex may represent opportunities for distinct efforts in further communications and evaluations by focusing informational and outreach efforts towards males and those who are not democratic-leaning to improve COVID-19 antiviral uptake.

Furthermore, within the 50−64 and ≥65 groups, the largest proportion of respondents who selected “Not Sure” or “Neither agree nor Disagree” was in response to knowledge of COVID-19 antivirals and taking antivirals if they tested positive. Among the ≥ 65 group, these respondents tended to be less likely to have higher education or to work full time. Among the 50−64 group, they tended to have higher incomes or to be less likely to be double boosted, while among the 50−64 and the group with immunosuppression or disability, they were less likely to be separated, divorced, or widowed. Additionally, the 12% of respondents in the 50−64 group who were not sure if they would take antivirals if their doctor recommended it were more likely to live in the Southern region and less likely to be fully vaccinated. These characteristics describe subpopulations of at-risk groups who could be targeted and who might benefit from specific messages to address perceptions about COVID-19 antivirals and possibly willingness to use them. Since characteristics vary by community, identifying multiple characteristics can be beneficial by allowing local entities and communities to identify local prevalent characteristics and focus their messaging and resources.

While some proportion of respondents in each age group were unsure about the effectiveness of antivirals and were neutral about taking them if they tested positive for COVID-19, far fewer respondents within each age group were unsure about taking antivirals if their doctor prescribed them. This reiterates prior findings that, despite personal attitudes and beliefs about health options, doctor’s recommendations are a potentially trusted source of information likely capable of overcoming individual patient concerns [41–43]. As such, this presents an opportunity for targeted interventions such as messaging and providing doctors with tools to strengthen the antiviral recommendations to at-risk groups of concern, and possibly increase antiviral use among at-risk groups for whom they are recommended.

### Perceived willingness to take antivirals

Willingness to take antivirals given a positive test was associated with being boosted or double boosted among 50–54 years, and those with immunosuppression or disability, findings similar to the characteristics associated with knowledge and attitudes toward antivirals [22]. Other socioeconomic factors indicating higher level of resources, consistent with the factors associated with knowledge and attitude, were also associated with willingness to take antivirals, such as working full time, owning a home, and education. Except among those ≥65 years, being fully vaccinated or boosted were also associated with being willing to take an antiviral if a doctor recommended it in each group.

Antivirals can reduce the risk of hospitalization, post-COVID conditions or Long COVID, and death [44], but less than 50% of those eligible in studies examining electronic medical record data are prescribed them [11], therefore the full potential life-saving use of antivirals is currently not being met. Provider-level barriers have been described [10], but less is understood about knowledge, attitudes, and perceptions of people who might get COVID-19 and might be eligible for treatment. This study illuminates some of the factors that are associated with knowledge of, attitudes toward, and willingness to take antivirals. While certain groups of people appear to have adequate knowledge some are willing to take an antiviral on the advice of a doctor, the consistent association with this willingness was previous vaccination.

### Strengths and limitations

The strengths of this study were the randomly selected participants, the oversampling for under-represented populations, the weighting to account for the U.S. population, and the multivariable analysis for certain outcomes. Limitations included first, that specific antivirals were not named in the questionnaire. Second, that specific underlying medical conditions were not solicited nor used to define immunocompromised or disability status. While age is the largest risk factor for severe COVID-19 outcomes, and we did have self-reported information about people with disability and immunosuppression, because the goal was a rapid assessment of knowledge, attitudes, and willingness, and the questionnaire was limited to 5 minutes to complete, we could not address knowledge, attitudes, and perceptions among people with specific medical conditions. Third, since state of disability was not asked without reference to ability to work, it is possible that any nuances among people with a disability who were able to work to some degree were not captured leading to possible under-ascertainment of disability and uncertainty in the estimates about this group’s knowledge, attitudes, and perceptions. Fourth, although we report the strongest predictors from the multivariable models, these variables accounted for less than half of the variance in each model, with some accounting for as little as 6.8% of the variance, although only predictors with at least 10% variance are highlighted, and low variance models can be valuable in highlighting significant associations [45]. Given that this is one of the first publicly available studies on this topic and one of the very few available to date, despite the variance levels, this study’s findings are valuable [45] to preliminary efforts to identify eligible populations who might be willing to use COVID-19 antivirals, while laying the ground work for more extensive studies that could account for a greater level of variance. Fifth, the data were self-reported and not confirmed through medical records and vaccination records. Sixth, some responses may have been influenced by social desirability bias and overestimated the level of knowledge, belief in antiviral effectiveness, and willingness to receive antivirals among the respondents. However, the extent of social desirability bias can be mitigated through study design, particularly by how data was collected [29,30]. The need to appear more favorable by overinflating positive behaviors may increase when there is a higher likelihood that the behavior will be linked to the respondent, such as when there is an interviewer present, in a group setting, or with linked identifiable information [29,46,47]. Minimizing that likelihood of being identified has been successful in mitigating this impact [29,30]. This study employed all three mitigation methods by utilizing an anonymous, self-administered survey which participants could take in privacy. While this likely reduced the amount of social desirability bias, there could be residual overestimation of desirable responses. Lastly, because this was a cross-sectional study, no causality can be inferred from the results, only significance of associations.

## Conclusion

Among adult respondents, most respondents were knowledgeable about antivirals and agreed that they can reduce the risk for severe illness and hospitalization, and were willing to take them, particularly if recommended by a doctor. However, groups that were less likely to have been vaccinated, those living in isolation, with lower incomes, lower levels of education, and who were not sure about whether they would take an antiviral or not, may be at risk for not asking for or receiving treatment to prevent severe COVID-19 outcomes. As such they represent a potential target group who might be more open to persuasive interventions to improve their COVID-19 antiviral uptake. Understanding factors associated with current attitudes, behaviors, and willingness to use antiviral medications for COVID-19 may help identify populations and targeted policies, messages, and interventions, such as advice from a trusted provider, to bridge the gap between public knowledge and available therapies and increase COVID-19 antiviral uptake. In a pandemic such as COVID-19, synergistic functioning between public health and private and public healthcare systems may ensure that patients and health care providers are aware and knowledgeable about available treatments for COVID-19, and that patients who are eligible for these lifesaving medicines receive them. As preparations for seasonal iterations of COVID-19 continue, these results may be applicable in developing targeted messaging to improve COVID-19 antiviral uptake among those who are eligible.
